# The Prevalence and Causes of Visual Impairment and Blindness Among Older Adults in the City of Lodz, Poland

**DOI:** 10.1097/MD.0000000000000505

**Published:** 2015-02-06

**Authors:** Michal S. Nowak, Janusz Smigielski

**Affiliations:** From the Department of Ophthalmology and Visual Rehabilitation (MSN), and the Department of Geriatrics (JS), Medical University of Lodz, Lodz, Poland.

## Abstract

To investigate the prevalence and causes of visual impairment and blindness in a sample of Polish older adults.

The study was designed in a cross-sectional and observational manner. Data concerning the vision status were assessed in 2214 eyes from 1107 subjects of European Caucasian origin; most of whom live in the city of Lodz, in central Poland. Visual impairment was defined as distance visual acuity <20/40 in the worse-seeing eye. Low vision was defined as best-corrected visual acuity (BCVA) <20/40 but >20/200 in better-seeing eye, and blindness was defined as BCVA ≤20/200 in both eyes (United States criteria).

Visual impairment was found in 27.5% subjects in the worse-seeing eye. Multiple regression analysis showed that increasing age (OR 0.98, 95% CI 0.97–0.99) and female gender (OR 1.47, 95% CI 1.11–1.93) were independent risk factors. No association was found between visual impairment and socioeconomic status of subjects. Noncorrectable visual impairment was found in 7.0% of subjects, including 5.2% of subjects with unilateral and 1.8% of subjects with bilateral visual impairment. Low vision and blindness accounted for 1.3% and 0.5%, respectively, and were only associated with older age (OR 1.05, 95% CI 1.02–1.10). Retinal diseases represented the major cause of noncorrectable visual impairment and accounted for more than half of causes of blindness.

Provision of appropriate refractive correction improves visual acuity in 75% subjects presenting with visual impairment. Retinal diseases are a major cause of noncorrectable visual impairment and blindness in this older population.

## INTRODUCTION

According to the latest reports of the World Health Organization (WHO), the total number of persons with visual impairment worldwide was estimated to be 285 million in 2010; of whom 39 million were blind. However, this number is less than the 314 million from previous reports. The differences in prevalence of visual impairment between high-income and low-income countries as well as between different WHO regions are still very large.^1–3^ On the basis of available reports, the rate of visual impairment among adults varies from 5% in Scandinavian countries to 46% in India (according to presenting vision).^4,5^ Despite the prevalence of visual impairment in all industrialized countries being rather low, the risk of age-related visual impairment in Europe is assumed to be on the rise, because of increased longevity.^6,7^

In the European region, the total number of persons with visual impairment is estimated to be more than 28 million; of whom 2.7 million are blind.^1^ According to the World Health Survey (WHS), which was carried out in adults in years 2002–2003 in 70 countries, the age-adjusted prevalence of any far vision difficulty in Europe varies from 5.7% in Norway to 25.2% in Russian Federation. The age-adjusted prevalence of severe or extreme far visual difficulty varies from 0.5% in Finland to 6.6% in Turkey, respectively.^1,2^

Although many studies concerning visual impairment have been conducted in Western European countries, there is a lack of studies from Eastern European nations (post-Soviet countries). From Poland there have been only few reports concerning children and we previously published a large data set acquired during a survey conducted on young men in the military population.^8–11^ Separate studies concerning older adults in Poland have not been undertaken. Taking this fact into account, we decided to perform another study concerning the population of citizens aged 35 years and older (see paragraph below for demography). The aim of this study was the assessment of the prevalence and causes of visual impairment and blindness in a sample population of Polish older adults. To the best of our knowledge, this is the first prevalence study from Poland concerning the visual status of the older population.

## MATERIAL AND METHODS

### Subjects

The study was designed in a cross-sectional and observational manner. Lodz voivodship, located in central Poland, is inhabited by 2.6 million people. The city of Lodz is the capital of the province and the second largest city in Poland. Lodz consists of 740,000 inhabitants (2011 national census), mostly of middle socioeconomic status.^12^ Because this study was a continuation of our previous survey, we used the same methodology for subject sampling. In brief “sample size for the study was calculated with 99% confidence, within an error bound of 5%. The sample size requirement was 661, as calculated by *n* = *Z*^2^ /4*d*^2^,

where *Z* = 2.57 for 99% confidence interval and *d* = 0.05 for 5% error bound. After allowing for an arbitrary 50% increase in sample size to accommodate possible inefficiencies associated with the sample design, the sample size requirement increased to 991 subjects.”^10,11^ We decided to define an older adult as a person of 35 years of age or older, because in our previous studies we considered young adult as person aged 18–34 years.^10,11^ Since the Department of Ophthalmology and Visual Rehabilitation of Medical University of Lodz has the biggest number of both outpatients and inpatients in the city, we decided to use simple systematic sampling to select our study population. In total, 14,110 patients were examined in our Outpatient Department in year 2012 and we included into the study every 10th subject aged 35 years and older. Based on age, the subjects were divided into two groups: group I aged 35–59 years and group II aged 60 years and older. The study was approved by the institutional review board of the Medical University of Lodz and patients’ data were all anonymized before any data analysis which included age socioeconomic status as well as brief details of the eye conditions. Because of the nature of the survey, the institutional review board waived the need for written informed consent from the participants, but otherwise the work was conducted in accordance with the provisions of the Declaration of Helsinki for research involving human subjects.

### Definitions and Eye Examinations

Distance visual acuity (VA) was tested monocularly using a retroilluminated Snellen chart at a distance of 4 m (with spectacles if worn). As part of the ophthalmic examination, cycloplegic refraction data were obtained in all subjects presenting with distance VA less than 20/40 in one or both eyes using the Topcon KR 8900 autorefractometer (supplied by Topcon Corporation, Tokyo, Japan). Based on this refraction, subjective refraction tests were performed to achieve best-corrected visual acuity (BCVA). Presenting visual impairment was defined as distance VA less than 20/40 (decimal VA, 0.5) in the worse-seeing eye. Correctable visual impairment was defined as that eliminated by refractive correction. Visual impairment, which could not be eliminated by refractive correction, was considered noncorrectable.^10^ We used United States criteria for the definitions of low vision and blindness.^13^ Low vision was defined as BCVA less than 20/40 (decimal VA, 0.5) but better than 20/200 (decimal VA, 0.1) in better-seeing eye. Blindness was defined as BCVA equal to or less than 20/200 (decimal VA, 0.1) in both eyes. Additionally, comprehensive eye examination included slit lamp and indirect ophthalmoscopic evaluation of the anterior and posterior segments, cover test, binocular vision and color vision assessments, intraocular pressure measurements, as well as other examinations, that is, visual field, ultrasound imaging, optical coherence tomography, fluorescein angiography, computed tomography (CT), and magnetic resonance imaging (MRI) when needed. The principal cause of impaired distance vision (VA <20/40 in worse eye) was assigned for each eye using a 14-item list. The cause of VA loss was assigned to refractive error in each eye, if VA improved to 20/40 or better with spectacle correction. For other causes of visual impairment and blindness we used the definition from 10th revision of International Statistical Classification of diseases, injuries, and causes of death (ICD-10).

### Data Management and Statistical Analysis

All statistical analyses were performed using STATISTICA v. 10.1 PL software (StatSoft Polska, Krakow, Poland). Prevalence rates of presenting correctable, noncorrectable visual impairment as well as of low vision and blindness were calculated. Prevalence rates of ocular causes of visual impairment and blindness were calculated. The associations between the distance VA and the subjects’ age and gender were explored by *χ*^2^ statistics (*P* < 0.05). Multiple logistic regression statistics were used to investigate the association of presenting visual impairment, low vision, and blindness with age, gender, and socioeconomic status of participants. Odds ratios (ORs) were computed. All sample means are reported with their standard errors. All presented confidence intervals (CIs) are 95% CI.

## RESULTS

### Subjects

The demographic characteristics of all participants in the study are presented in Table 1. A total of 1107 persons aged ≥35 years were enumerated and included into the study. According to 2011 national census, they were a fair representation of the population of the city of Lodz in terms of sex distribution (statistical analysis—chi-square test: *χ2* = 3.64, *P*  > 0.05) and socioeconomic status.^12^ The mean age of the study population was 60.4 ± 7.1 years (range, 35–97 years). Among all 1107 subjects, 642 were women (58.0%) and 465 were men (42.0%). They were divided into two age groups: 520 (47.0%) subjects were between 35 and 59 years of age, and 587 (53.0%) subjects were aged 60 years and older. Statistical analysis revealed that the two groups did not vary significantly in gender (*χ*^2^ test *P* = 0.158). However, in our study women outnumbered men by 16.0% and this was mainly attributable to excess male death rate characteristic of the Soviet areas, which is still producing its effect in Poland.^12^

**TABLE 1:**
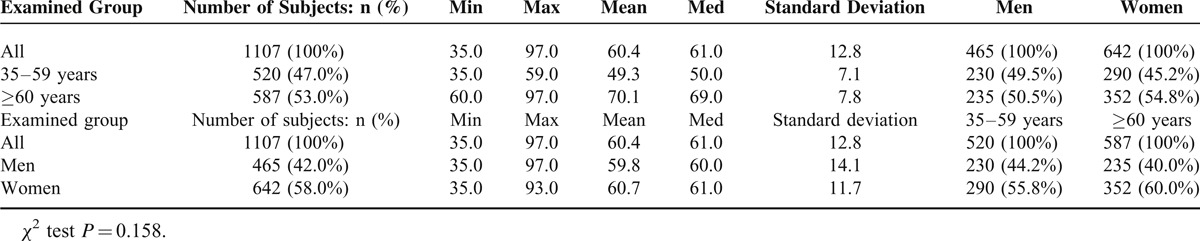
The Patient Grouping as a Function of Age

### Presenting Visual Impairment

We obtained reliable VA measurements in 2214 eyes of 1107 subjects (Table 2). Overall 42.0% (95% CI 39.1–44.9) subjects had normal vision, that is, distance VA of ≥40/50 in both eyes, and 30.5% (95% CI 27.8–33.2) subjects had near normal vision, that is, distance VA <40/50 but ≥20/40 in worse-seeing eye. Presenting visual impairment, that is, distance VA of <20/40 in worse-seeing eye was found in 27.5% (95% CI 24.8–30.1) subjects. Differences between distance VA in particular age groups as well as among genders were statistically significant (*χ*^2^ test *P* < 0.001 and *P* = 0.01, respectively). The prevalence of distance VA of ≥40/50 in both eyes was lower, and the prevalence of distance VA of <20/40 in the worse-seeing eye was higher in the age group ≥60 years and in women. Multiple regression analysis showed that the prevalence of presenting visual impairment for a 20/40 cutoff in worse-seeing eye was associated with age (OR 0.98, 95% CI 0.97–0.99) and with female gender (OR 1.47, 95% CI 1.11–1.93). For each year increase of subject's age, the odds of presenting visual impairment increased by 2%. However, no association was found between presenting visual impairment and socioeconomic status of subjects.

**TABLE 2:**
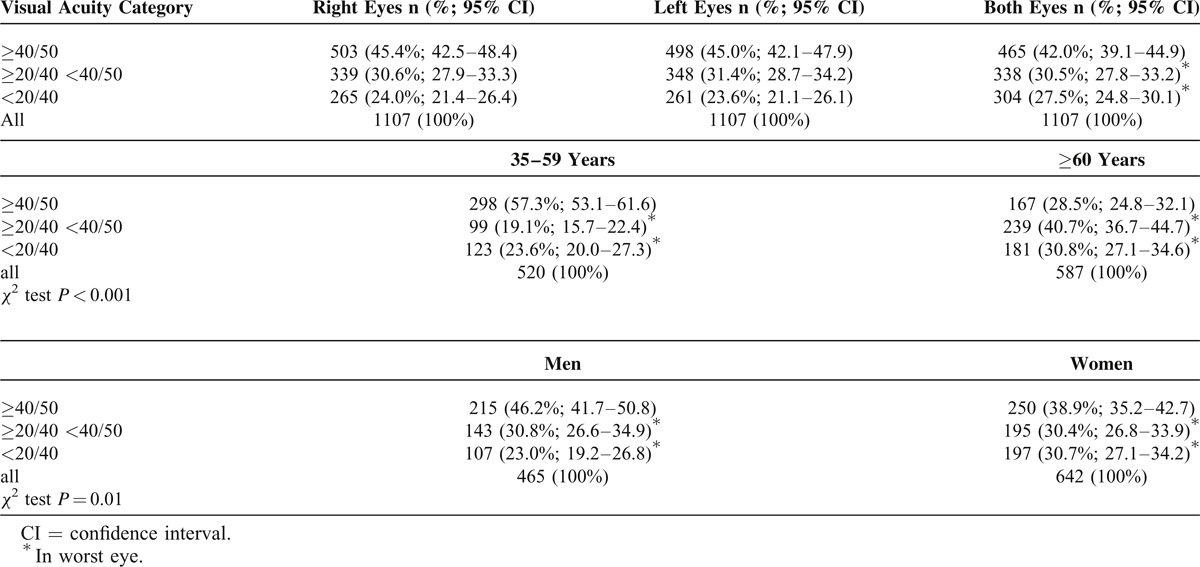
Distribution of Distance Visual Acuity and Presenting Visual Impairment

### Correctable and Noncorrectable Visual Impairment

The prevalence of correctable and noncorrectable visual impairment is presented in Table 3. Subjective refraction measurements were performed in all eyes with distance VA of <20/40. In total, presenting visual impairment was found in 304 (27.5%; 95% CI 24.8–30.1) subjects in worse-seeing eye. Of these, distance vision could be improved in 227 (20.5%; 18.1–22.9) subjects, after subjective refraction. Overall 77 (7.0%; 95% CI 5.5–8.4) subjects had noncorrectable visual impairment in worse-seeing eye. Of them 57 (5.2%, 95% CI 3.8–6.4) subjects had unilateral visual impairment. Twenty patients had bilateral visual impairment (BCVA <20/40 in both eyes), which accounted for 1.8% (95% CI 1.0–2.6) of whole population. This included participants with both low vision and blindness. The prevalence of low vision and blindness was 1.3% (95% CI 0.6–1.9) and 0.5% (95% CI 0.1–1.0), respectively. Since the number of blind people was low, we combined two groups (low vision and blindness) together for analysis. The number of participants with low vision and blindness was higher in the age group ≥60 years and in women. However, in multiple logistic regression modeling with age, gender, and socioeconomic status, low vision and blindness were only associated with older age (OR 1.05, 95% CI 1.02–1.10). Gender and socioeconomic status were not significant factors.

**TABLE 3:**
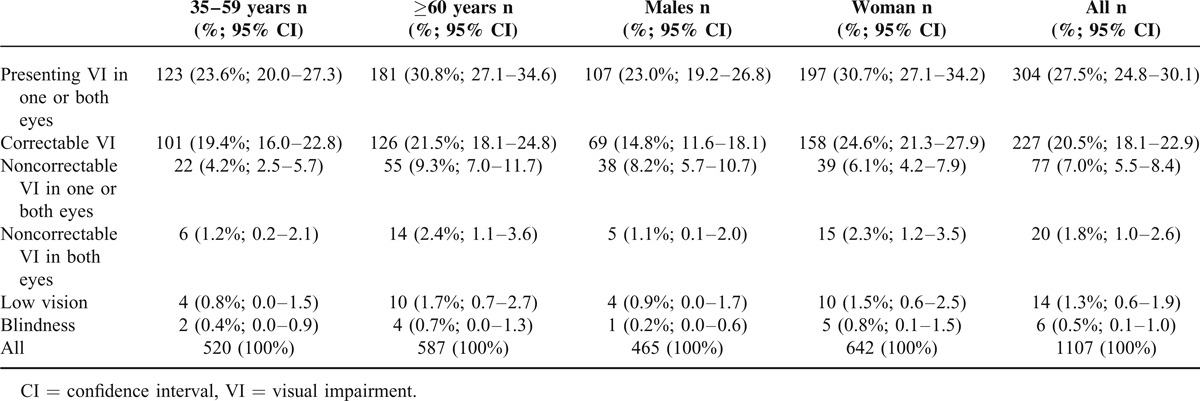
The Prevalence of Correctable and Noncorrectable Visual Impairment in the Examined Group

### The Principal Causes of Unilateral and Bilateral Visual Impairment

Six subjects were classified as “blind” (BCVA ≤20/200 in both eyes). This was attributable to one case each of age-related macular degeneration (AMD), glaucoma, degenerative myopia, corneal problems, toxoplasmosis, and ocular albinism (both classified as other retinal disorders). Table 4 shows the distribution and prevalence of unilateral and bilateral noncorrectable visual impairment. Overall AMD was the leading cause of noncorrectable visual impairment accounted for 18.2% of all visual impairment cases, followed by cataract and amblyopia both accounted for 15.6%. Statistical analysis of the differences between the prevalence of particular causes of noncorrectable visual impairment and the subjects’ age as well as gender was not performed due to the low number of cases. However, in younger individuals (35–59 years) amblyopia was the main cause, followed by diabetic retinopathy and corneal problems. In older individuals (≥60 years) AMD, followed by cataract and glaucoma, was the main cause. A comparison of causes of noncorrectable visual impairment between genders showed that in both sexes AMD was the most common cause found. For unilateral noncorrectable visual impairment, amblyopia was responsible for 21.0 % cases, followed by AMD and cataract. AMD was the leading cause of bilateral noncorrectable visual impairment and accounted for 25.0% cases, followed by cataract and glaucoma. When retinal disorders were grouped together, they represented the most common cause of both unilateral and bilateral noncorrectable visual impairment.

**TABLE 4:**
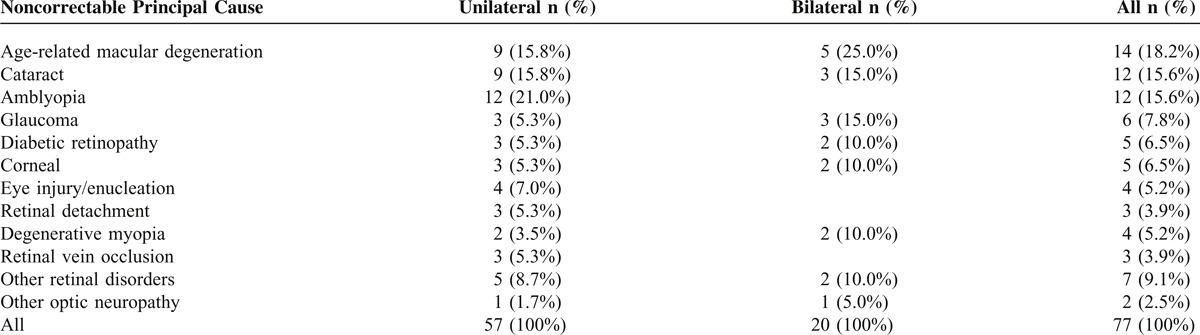
The Principal Causes of Unilateral and Bilateral Noncorrectable Visual Impairment

## DISCUSSION

This study on the outpatient referrals to our eye hospital describes a large unselected population of Polish citizens and provides reliable data about the prevalence and causes of visual impairment in older adults in the City of Lodz, in central Poland. According to WHO, low vision was defined as BCVA of less than 6/18 but equal to or better than 3/60 in better-seeing eye, and blindness was defined as BCVA of less than 3/60 in both eyes.^1^ However, in recent years, most researchers conducting population-based studies have in practice departed from the WHO definitions in two aspects. “First, they have recorded presenting vision (habitual vision), that is, the VA of the patients as they come to the examination site. Second, the cutoff level of acuity for visual impairment had been moved to less than 6/12 (20/40) in recognition of the increasing visual demands in all countries, for example, for driving and use of computers.”^13^ Since VA of 20/40 is a required criterion for driving license certification in many countries including Poland, our data have been presented using United States criteria for the definitions of low vision and blindness. This methodology is similar to that in earlier population based surveys in Europe, Australia, United States, and elsewhere.^14–20^ Our data have been also presented based on presenting VA in worse-seeing eye to show the real magnitude of visual impairment in this older population. Overall the prevalence of presenting visual impairment in the examined population was rather high (27.5%). The study revealed that presenting visual impairment was significantly associated with older age and with female gender. Our prevalence rate of presenting visual impairment was similar to some rates found in a number of population-based studies from low-income south Asian countries,^5,21–23^ and was higher than in several previous population-based surveys from Europe, Australia, both Americas, and Singapore.^4,15,18,24–26^ But those studies defined presenting visual impairment as VA in a better-seeing eye. Direct comparison of our results to the results obtained in other studies conducted on adults is also limited due to differences in study design and population sampling. The major limitation is the fact that we enrolled patients solely from our Outpatients Department, thus the prevalence of ocular disorders might be overestimated. However, our results were in agreement with the research performed in Hong Kong by Michon et al, who used similar criteria and found presenting visual impairment in at least one eye in 41.3% of subjects 60 years of age and older.^27^ Hong Kong is also a middle-income country, similar to Poland. We did not find any association between presenting visual impairment and socioeconomic status of subjects. In total, 27.5% of subjects participating in our study had presenting visual impairment in at least one eye, as mentioned earlier. But the prevalence of visual impairment in either eye, not due to refractive error, was only 7.0%. We found that in 75% of the cases, based on the presenting vision, visual impairment could be simply eliminated with refraction and appropriate vision correction. Findings from our study were in agreement with research on the correctable visual impairment of older adults in Australia, predominantly of European Caucasian origin, where the rate of correctable visual impairment was 68%,^26^ and with data from the 2005–2008 National Health and Nutrition Examination Survey (NHANES) in the United States, where around 73% of subjects with presenting visual impairment could achieve good VA with correction.^15^ The present study revealed that bilateral visual impairment (BCVA <20/40 in both eyes) accounted for 1.8% of whole population. This included participants with both low vision (1.3%) and blindness (0.5%). Comparison of sampling techniques and the prevalence of low vision and blindness in different populations from previously published studies is presented in Table 5. Multiple regression analysis showed that low vision and blindness were only associated with age. Our data also indicated that distribution of causes of noncorrectable visual impairment varied by age. Among persons aged 35 to 59 years amblyopia was the main cause, followed by diabetic retinopathy and corneal problems. In older individuals (≥60 years) AMD, followed by cataract and glaucoma, was the main cause. In the whole population, AMD was the leading cause of noncorrectable visual impairment followed by cataract and amblyopia. Overall retinal diseases represented the major cause of both unilateral and bilateral noncorrectable visual impairment and accounted for two-thirds (66.6%) of the causes of bilateral blindness. Our results were in agreement with other studies from Europe, Israel, and Australia, which found retinal diseases as a major cause of bilateral blindness in subjects of predominantly European Caucasian origin.^6,14,28–32^

**TABLE 5:**
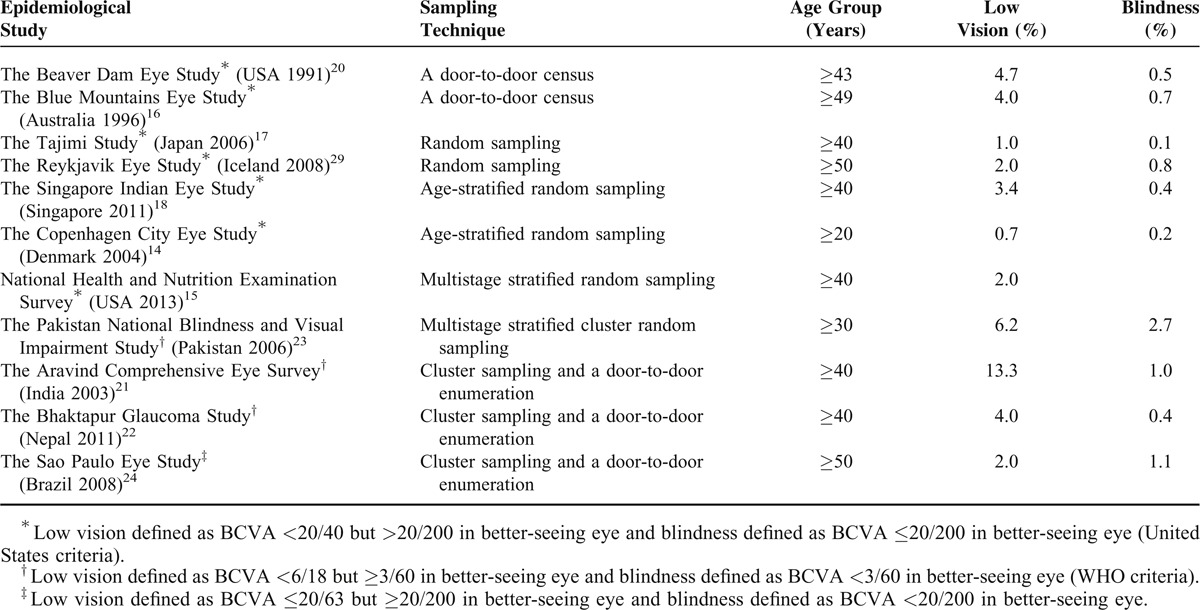
Comparison of Sampling Techniques and the Prevalence of Low Vision and Blindness in Different Populations from Previously Published Studies

In conclusion, the present study reports for the first time an analysis of visual status of older adults in Poland. Increasing age and female sex are independent risk factors associated with visual impairment. Provision of appropriate refractive correction improves VA in 75% subjects with presenting visual impairment. Retinal diseases are a major cause of noncorrectable visual impairment and blindness, and are likely to become more important due to an ageing population.

## References

[1] Pascolini D Mariotti SP Global estimates of visual impairment: 2010. *Br J Ophthalmol* 2012; 96:614–618. 22133988 10.1136/bjophthalmol-2011-300539

[2] Freeman EE Roy-Gagnon MH Samson E The global burden of visual difficulty in low, middle and high income countries. *PLoS One* 2013; 8:e63315. 23675477 10.1371/journal.pone.0063315 PMC3651198

[3] Resnikoff S Pascolini D Mariotti SP Global magnitude of visual impairment caused by uncorrected refractive errors in 2004. *Bull World Health Organ* 2008; 86:63–70. 18235892 10.2471/BLT.07.041210 PMC2647357

[4] Laitinen A Laatikainen L Harkanen T Prevalence of major eye diseases and causes of visual impairment in the adult Finnish population: a nationwide population- based survey. *Acta Ophthalmol* 2010; 88:463–471. 19878108 10.1111/j.1755-3768.2009.01566.x

[5] Murthy GV Vashist P John N Prevalence and causes of visual impairment and blindness in older adults in an area of India with a high cataract surgical rate. *Ophthalmic Epidemiol* 2010; 17:185–195. 20642340 10.3109/09286586.2010.483751 PMC6031136

[6] Skaat A Chetrit A Belkin M Time trends in the incidence and causes of blindness in Israel. *Am J Ophthalmol* 2012; 153:214–221. 22264945 10.1016/j.ajo.2011.08.035

[7] Wolfram C Pfeiffer N Blindness and low vision in Germany 1993–2009. *Ophthalmic Epidemiol* 2012; 19:3–7. 22273353 10.3109/09286586.2011.628136

[8] Pieczyrak D Miśkowiak B Condition of the visual system and school achievements in 6 to 10 years old children from Wielkopolska region as detected by visual screening and questionnaire studies. *Klin Oczna* 2011; 113:243–248. 22256566

[9] Czepita D Zejmo M Mojsa A Prevalence of myopia and hyperopia in a population of Polish schoolchildren. *Ophthalmic Physiol Opt* 2007; 27:60–65. 17239191 10.1111/j.1475-1313.2006.00419.x

[10] Nowak MS Goś R Jurowski P Correctable and non-correctable visual impairment among young males: a 12-year prevalence study of the Military Service in Poland. *Ophthal Physiol Opt* 2009; 29:443–448. 10.1111/j.1475-1313.2008.00628.x 19292830

[11] Nowak MS Jurowski P Goś R Ocular findings among young men: a 12 year prevalence study of military service in Poland. *Acta Ophthamol* 2010; 88:535–540. 10.1111/j.1755-3768.2008.01476.x 19456312

[12] The National Census of Population and Housing 1 April – 30 June 2011. Warszawa (2013) Zakład Wydawnictw Statystycznych http://stat.gov.pl/cps/rde/xbcr/gus/LUD_ludnosc_stan_str_dem_spo_NSP2011.pdf.

[13] Johnson GJ Minassian DC Weale RA The Epidemiology of Eye Disease. 3rd ed. 2012; London: Imperial College Press, 3–61.

[14] Buch H Vinding T la Cour M Prevalence and causes of visual impairment and blindness among 9980 Scandinavian adults: the Copenhagen City Eye Study. *Ophthalmology* 2004; 111:53–61. 14711714 10.1016/j.ophtha.2003.05.010

[15] Chou CF Cotch MF Vitale S Age-related eye diseases and visual impairment among U.S. adults. *Am J Prev Med* 2013; 45:29–35. 23790986 10.1016/j.amepre.2013.02.018 PMC4072030

[16] Attebo K Mitchell P Smith W Visual acuity and the causes of visual loss in Australia. The Blue Mountains Eye Study. *Ophthalmology* 1996; 103:357–364. 8600410 10.1016/s0161-6420(96)30684-2

[17] Iwase A Araie M Tomidokoro A Prevalence and causes of low vision and blindness in a Japanese adult population: the Tajimi Study. *Ophthalmology* 2006; 113:1354–1362. 16877074 10.1016/j.ophtha.2006.04.022

[18] Zheng Y Lavanya R Wu R Prevalence and causes of visual impairment and blindness in an urban Indian population: the Singapore Indian Eye Study. *Ophthalmology* 2011; 118:1798–1804. 21621261 10.1016/j.ophtha.2011.02.014

[19] You QS Xu L Yang H Five-year incidence of visual impairment and blindness in adult Chinese: the Beijing Eye Study. *Ophthalmology* 2011; 118:1069–1075. 21211839 10.1016/j.ophtha.2010.09.032

[20] Klein R Klein BE Linton KL The Beaver Dam Eye Study: visual acuity. *Ophthalmology* 1991; 98:1310–1315. 1923372 10.1016/s0161-6420(91)32137-7

[21] Thulasiraj RD Nirmalan PK Ramakrishnan R Blindness and vision impairment in a rural south Indian population: the Aravind Comprehensive Eye Survey. *Ophthalmology* 2003; 110:1491–1498. 12917162 10.1016/S0161-6420(03)00565-7

[22] Thapa SS Berg RV Khanal S Prevalence of visual impairment, cataract surgery and awareness of cataract and glaucoma in Bhaktapur district of Nepal: the Bhaktapur Glaucoma Study. *BMC Ophthalmol* 2011; 11:2. 21255382 10.1186/1471-2415-11-2 PMC3036669

[23] Jadoon MZ Dineen B Bourne RR Prevalence of blindness and visual impairment in Pakistan: The Pakistan National Blindness and Visual Impairment Survey. *Invest Ophthalmol Vis Sci* 2006; 47:4749–4755. 17065483 10.1167/iovs.06-0374

[24] Salomao SR Cinoto RW Berezovsky A Prevalence and causes of low vision and blindness in older adults in Brazil: the São Paulo Eye Study. *Ophthalmic Epidemiol* 2008; 15:167–175. 18569812 10.1080/09286580701843812 PMC6031128

[25] Limburg H Barria von-Bischhoffshausen F Gomez P Review of recent surveys on blindness and visual impairment in Latin America. *Br J Ophthalmol* 2008; 92:315–319. 18211928 10.1136/bjo.2007.125906

[26] Foran A Rose K Wang JJ Correctable visual impairment in an older population: the Blue Mountains Eye Study. *Am J Ophthalmol* 2002; 134:712–719. 12429248 10.1016/s0002-9394(02)01673-2

[27] Michon JJ Lau J Chan WS Prevalence of visual impairment, blindness and cataract surgery in the Hong Kong elderly. *Br J Ophthalmol* 2002; 86:133–139. 11815334 10.1136/bjo.86.2.133 PMC1771025

[28] Gunnlaugsdottir E Arnarsson A Jonasson F Prevalence and causes of visual impairment and blindness in Icelanders aged 50 years and older: the Reykjavik Eye Study. *Acta Ophthalmol* 2008; 86:778–785. 18513265 10.1111/j.1755-3768.2008.01191.x

[29] Finger RP Fimmers R Holz FG Incidence of blindness and severe visual impairment in Germany: projections for 2030. *Invest Ophthalmol Vis Sci* 2011; 52:4381–4389. 21447690 10.1167/iovs.10-6987

[30] Foran S Wang JJ Mitchell P Causes of visual impairment in two older population cross-sections: The Blue Mountains Eye Study. *Ophthalmic Epidemiol* 2003; 10:215–225. 14628964 10.1076/opep.10.4.215.15906

[31] Cruciani F Amore F Albanese G Investigation about causes of blindness and low vision among members of Blind and Visually Impaired Italian Union (UICI). *Clin Ter* 2011; 162:e35–e42. 21533307

[32] Klaver CC Wolfs RC Vingerling JR Age-specific prevalence and causes of blindness and visual impairment in an older population: the Rotterdam Study. *Arch Ophthalmol* 1998; 116:653–658. 9596502 10.1001/archopht.116.5.653

