# The Prevalence and Causes of Visual Impairment and Blindness Among Older Adults in the City of Lodz, Poland: Erratum

**DOI:** 10.1097/01.md.0000464212.78544.f2

**Published:** 2015-02-20

In the article “The Prevalence and Causes of Visual Impairment and Blindness Among Older Adults in the City of Lodz, Poland”,^1^ which appeared in Volume 94, Issue 5 of *Medicine*, the authors’ affiliation line was omitted. The affiliation line should read as follows:

From the Department of Ophthalmology and Visual Rehabilitation (MSN), and the Department of Geriatrics (JS), Medical University of Lodz, Lodz, Poland.

The article has since been corrected online.
